# Research and Application of Artificial Intelligence Based on Electronic Health Records of Patients With Cancer: Systematic Review

**DOI:** 10.2196/33799

**Published:** 2022-04-20

**Authors:** Xinyu Yang, Dongmei Mu, Hao Peng, Hua Li, Ying Wang, Ping Wang, Yue Wang, Siqi Han

**Affiliations:** 1 Division of Clinical Research The First Hospital of Jilin University Changchun China; 2 Department of Medical Informatics School of Public Health Jilin University Changchun China

**Keywords:** electronic health records, artificial intelligence, neoplasms, machine learning

## Abstract

**Background:**

With the accumulation of electronic health records and the development of artificial intelligence, patients with cancer urgently need new evidence of more personalized clinical and demographic characteristics and more sophisticated treatment and prevention strategies. However, no research has systematically analyzed the application and significance of artificial intelligence based on electronic health records in cancer care.

**Objective:**

The aim of this study was to conduct a review to introduce the current state and limitations of artificial intelligence based on electronic health records of patients with cancer and to summarize the performance of artificial intelligence in mining electronic health records and its impact on cancer care.

**Methods:**

Three databases were systematically searched to retrieve potentially relevant papers published from January 2009 to October 2020. Four principal reviewers assessed the quality of the papers and reviewed them for eligibility based on the inclusion criteria in the extracted data. The summary measures used in this analysis were the number and frequency of occurrence of the themes.

**Results:**

Of the 1034 papers considered, 148 papers met the inclusion criteria. Cancer care, especially cancers of female organs and digestive organs, could benefit from artificial intelligence based on electronic health records through cancer emergencies and prognostic estimates, cancer diagnosis and prediction, tumor stage detection, cancer case detection, and treatment pattern recognition. The models can always achieve an area under the curve of 0.7. Ensemble methods and deep learning are on the rise. In addition, electronic medical records in the existing studies are mainly in English and from private institutional databases.

**Conclusions:**

Artificial intelligence based on electronic health records performed well and could be useful for cancer care. Improving the performance of artificial intelligence can help patients receive more scientific-based and accurate treatments. There is a need for the development of new methods and electronic health record data sharing and for increased passion and support from cancer specialists.

## Introduction

### Overview

Cancer is known as one of the greatest challenges in health care, and its burden has risen in recent years, calling for a better understanding of clinical prediction strategies in real patient populations. Electronic health records (EHRs) integrate true information about patient care, such as demographics, medical history, and insurance [1]. The secondary use of EHRs is opening immense research avenues and opportunities for improving cancer management. However, there are many challenges of the secondary use of EHRs, and much valuable information is locked behind these vast amounts of complex data. Artificial intelligence (AI) techniques and methods are believed to be the most critical tool to alleviate this issue. Further, an increasing amount of data available in EHRs provides a new environment for the application of AI [2]. With the help of AI-based EHRs, each patient with cancer is more likely to be treated according to the best available knowledge, which is constantly updated for the benefit of the next patient, thereby improving clinical decision-making [3,4]. Despite the rapid development of technology, significant challenges remain to obtain valuable information quickly and accurately based on EHRs to better inform clinical decision-making.

### Objectives

The aim of this study was to conduct a review to introduce the current state and limitations of AI based on EHRs from patients with cancer and to explore the opportunities and challenges in this field. The objectives were to review the aspects of categorization of neoplasms, methods and algorithms, and applications in the field of cancer care, EHR data and data sets. These aspects were analyzed to summarize the performance of AI in mining EHRs and its impact on cancer care.

## Methods

### Search Strategy

The Web of Science Core Collection, PubMed, and the Association for Computing Machinery Digital Library databases were systematically searched to extract potentially relevant papers published from January 2009 to October 2020. The search expression was designed around 3 concepts: AI, cancer, and EHRs. They were combined using the AND Boolean operator. The Web of Science Core Collection search included the following terms, which were selected by referring to the entry terms of Medical Subject Headings and translated for the other databases. The English language was used as an additional filter.

1. AI: AI OR artificial intelligence OR natural language processing OR NLP OR natural language understanding OR NLU OR machine learning OR deep learning OR neural network OR support vector machine OR prediction network OR forecast model OR data mining OR supervised learning OR time series prediction OR intelligence, artificial OR computational intelligence OR intelligence, computational OR machine intelligence OR intelligence, machine OR computer reasoning OR reasoning, computer OR computer vision system OR system, computer vision
2. EHRs: EMR OR electronic medical records OR EHR OR electronic human records OR medical record, electronic OR health record, electronic OR medical record, computerized OR computerized medical record.
3. Cancer: cancer OR oncology OR tumor OR neoplasm OR neoplasia OR tumor OR malignancy

### Study Selection

We followed the PRISMA (Preferred Reporting Items for Systematic reviews and Meta-Analyses) guidelines [5]. The abstracts and titles were independently evaluated by 2 reviewers (XY and HP). Two reviewers (XY and PW) independently reviewed the full texts. Reviewers resolved disagreements by reaching consensus and consulted HL after group discussion if they held different opinions. Papers were included in this review if they met the following criteria: (1) peer-reviewed studies only, (2) the studies were on patients with cancer or on solving cancer problems, (3) the research methods used AI, (4) the study data were EHRs and the purpose of the paper was not to build an electronic medical record system, (5) a journal paper or a proceeding paper, (6) a research paper and not a review (including systematic review, meta-analysis, etc), and (7) published in the English language. All reviewers had medical informatics expertise; a basic understanding of EHRs, AI, and cancer; and strict adherence to the inclusion criteria.

### Data Collection Process and Data Items

The included papers were cited in an Excel spreadsheet by the reviewers. Reviewers agreed in a group meeting on what to look for in full-texts. According to the research objectives, we retrieved the following data from the key information: study details (including title, author, journal, time of publication), EHR details (including data period, data type, number of sources of data, data set size, data set publicly available, language, patient sample size), AI details (including algorithm categories, precision, negative predictive value, sensitivity [recall], specificity, F-score, accuracy, area under the curve [AUC], and applications), and cancer category. The notes were discussed in a consensus meeting between 2 reviewers after they independently retrieved the detailed data about the items, and they were asked to identify possible bias [6,7] in each paper. Publication bias, unblinded trial bias, and time lag bias were identified. No paper was discarded because of bias. The summary measures used in this analysis were the number and frequency of occurrence of the themes identified by the reviewers. Owing to the heterogeneity in the population, index method [8], and outcomes, we did not perform a quantitative synthesis of the results.

## Results

### Search and Selection Results

**Figure 1:**
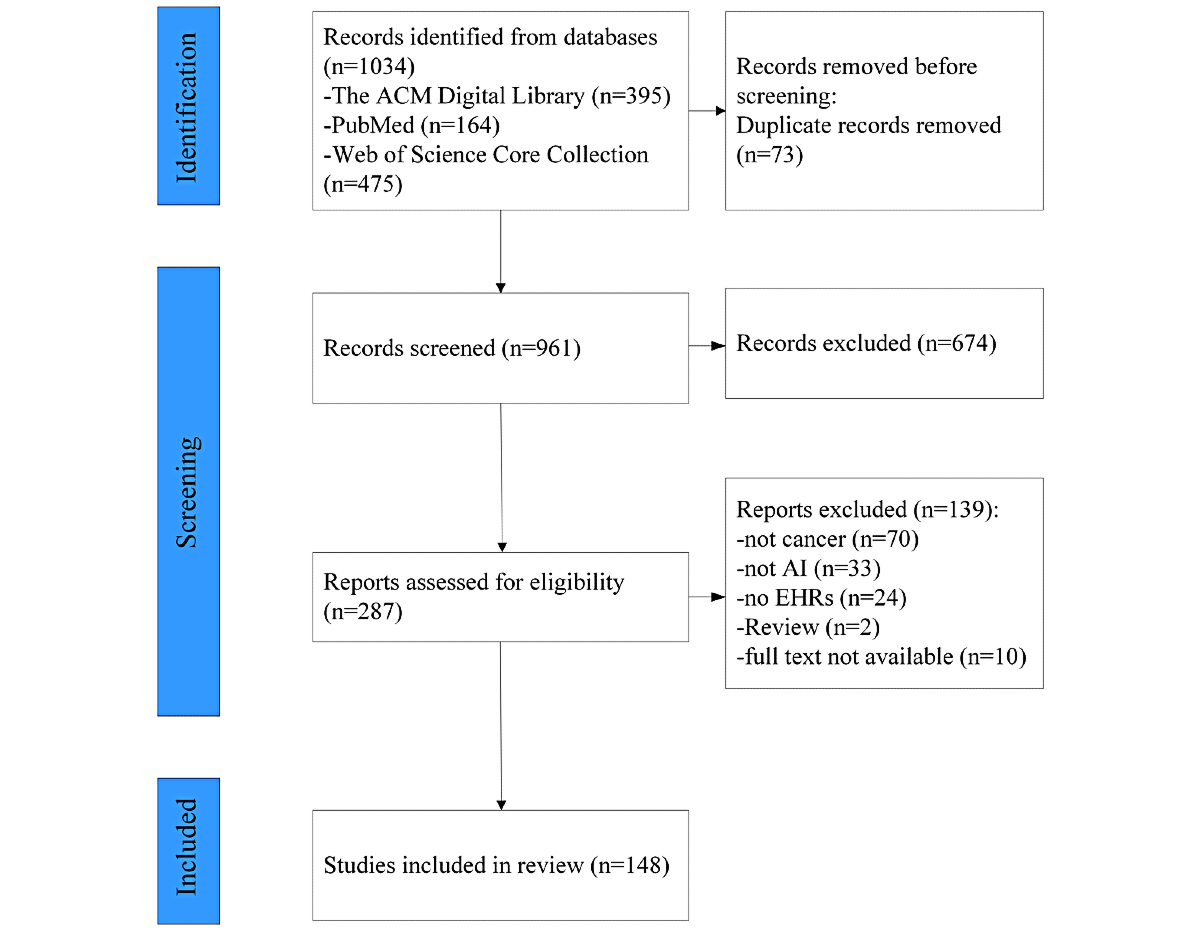
Paper selection flowchart. ACM: Association for Computing Machinery; AI: artificial intelligence; EHR: electronic health record.

A total of 1034 papers were initially retrieved, with 395 papers from the Association for Computing Machinery Digital Library, 164 from PubMed, and 475 from Web of Science Core Collection; 674 were removed after scanning the titles and abstracts and after removing 73 duplicates; and 287 papers were ultimately identified for full-text review. Following screening and eligibility, 148 papers were included in the final review. The flowchart of the selection process is presented in Figure 1. The most common reasons for exclusion were as follows: (1) the paper was not directly related to cancer (n=346), (2) the paper was a review and neither a journal paper nor a proceeding paper (n=256), (3) the paper was not based on EHRs (n=134), and (4) the research methods did not incorporate AI (n=67). The observations from each paper are summarized in the spreadsheet shown in Multimedia Appendix 1.

### Categorization of the Neoplasms

**Figure 2:**
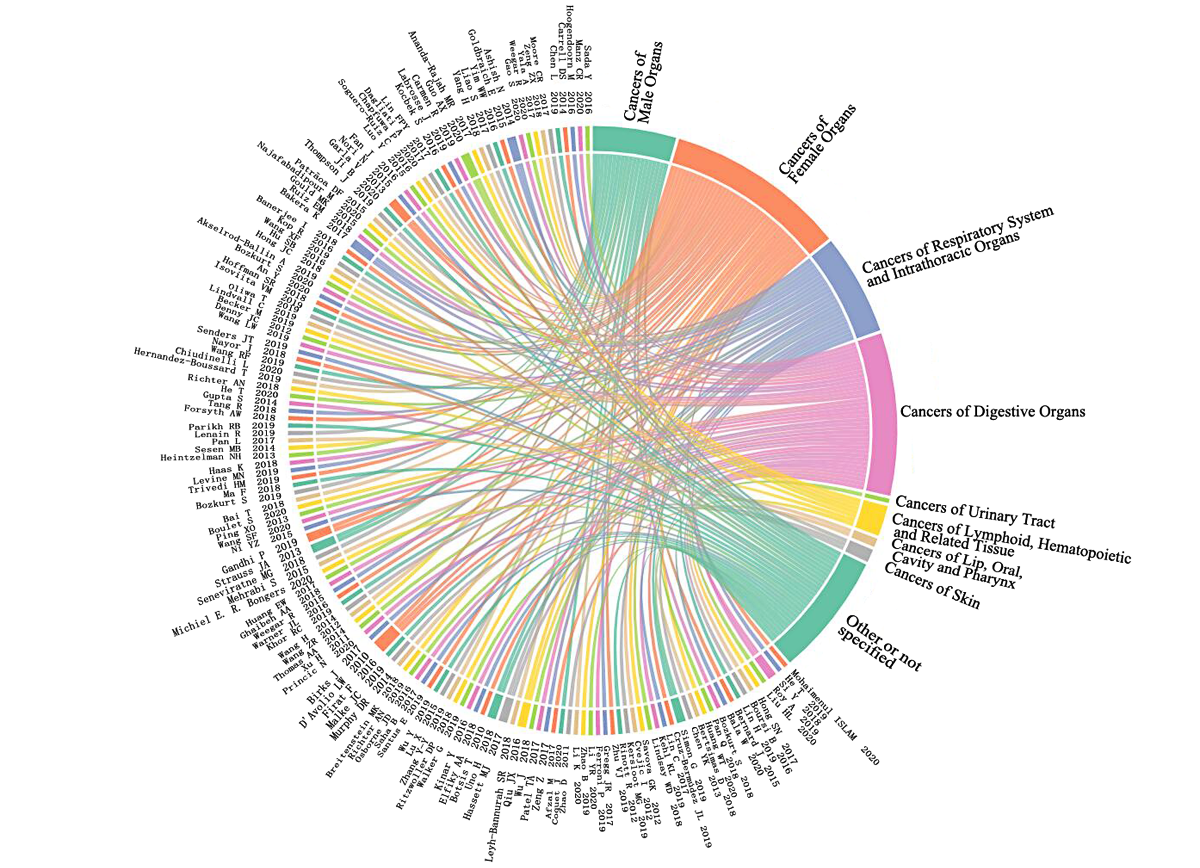
Relationship between the categorizations of the neoplasms and the papers included in this review (the complete reference details of the papers cited in this figure are provided in Multimedia Appendix 1).

The diseases studied in the 148 papers could be grouped into 9 unique categories of neoplasms according to the anatomical site of the lesion and International Classification of Diseases, tenth revision. The 3 most studied cancer categories were (1) cancers of female organs (n=42), (2) cancers of digestive organs (n=38), and (3) cancers of the respiratory system and intrathoracic organs (n=23). The relationship between each paper and the cancers studied is shown in Figure 2. The complete reference details of the papers cited in Figure 2 are provided in Multimedia Appendix 1. Most of the works on cancers of female organs focused on breast cancer. Receptor status phenotypes, biomarker status, and frequent patterns of care were obtained from EHRs of patients with breast cancer by using AI. For cancers of digestive organs, the types of cancers studied were relatively diverse, mainly comprising colorectal cancer (CRC) and liver cancer types. Earlier detection of CRC attracted the greatest attention from researchers. Because CRC symptoms develop slowly and insidiously over years, early diagnosis offers great opportunity to improve outcomes [9]. AI was constructed to identify the risk of CRC based on demographic and behavioral factors, analysis of complete blood counts [10], and so on. Clinically relevant features of liver cancer were extracted from EHRs, such as tumor reference resolution, tumor number, and largest tumor sizes [11]. Lung cancer was the only cancer of the respiratory system and intrathoracic organs studied in the papers we investigated. For example, a Lung Cancer Assistant was designed to provide decision support for experts in lung cancer multidisciplinary teams [12].

### Methods and Algorithms

#### Machine Learning Algorithms

**Figure 3:**
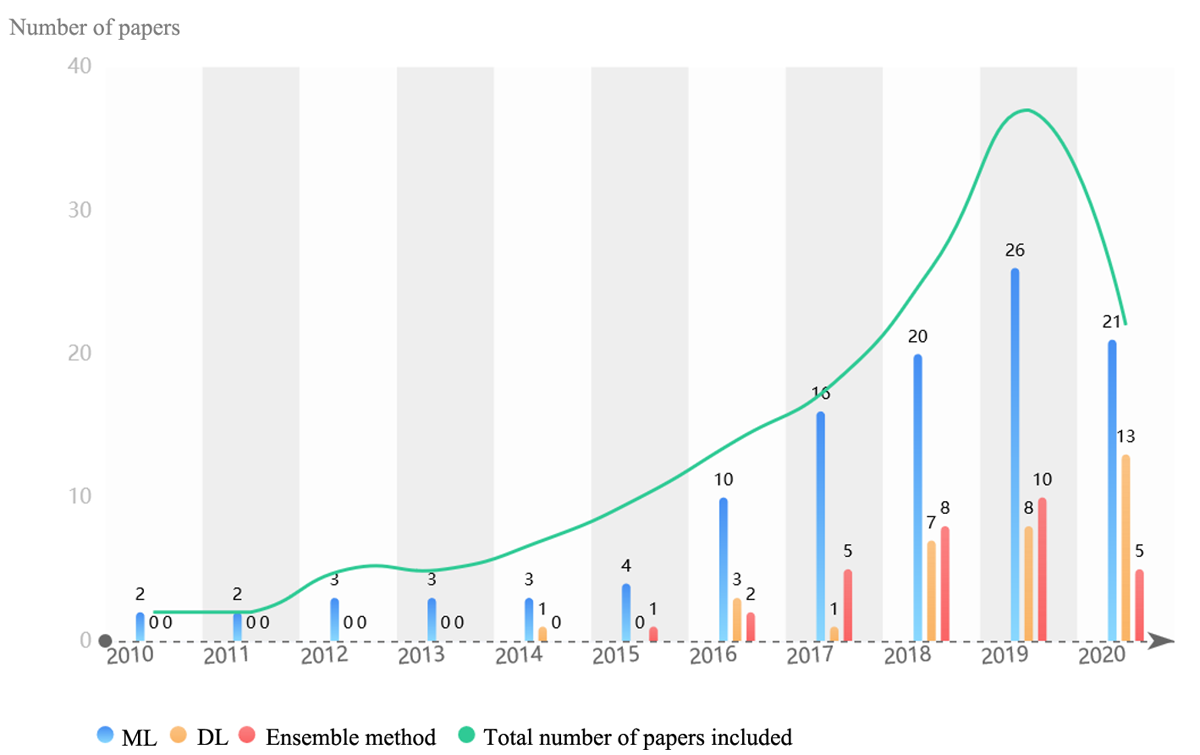
Machine learning algorithms for cancer. DL: deep learning; ML: machine learning.

Machine learning (ML) is an important way to achieve AI. A total of 110 papers used ML algorithms, among which support vector machine (SVM) (n=29) and logistic regression (n=28) were the most commonly used. SVM works well for data sets that are not linearly separable or highly unbalanced, which is important for EHR analysis. Several studies combined SVM with natural language processing (NLP) to extract breast cancer, CRC, and other cancer information from EHRs [13,14]. Logistic regression has been improved to the level of a more sophisticated algorithm for EHR mining of cancer patient data and combined with the lasso penalty [15], a convolutional neural network [16], and other methods in recent years. These algorithms are simple insightful white-box classification algorithms with advantages in interpretability [17] and sensitivity of data details [18]. In fact, these single-model methods were rarely used independently for prediction but used as a baseline to compare the performance of new technologies and methods. However, the deep learning (DL) algorithm and ensemble methods are increasing rapidly (as shown in Figure 3). The ensemble method (n=31), a single strong model combined with multiple weak models, showed high accuracy in processing EHRs. Gradient boosting and random forest performed better than SVM, decision tree, and lasso in classifying free-text pathology reports for prostate cancer into stage groups and identifying cases of metastatic prostate cancer [19,20]. DL (n=33) demonstrated great performance in cancer domains as well. Gao et al [21] designed a modular component with recurrent neural network, including long short-term memory and gated recurrent units for capturing case-level context, to improve the classification accuracy of aggregate-level labels for cancer pathology reports. Recurrent neural network was designed particularly to deal with temporal data, which is very promising for EHRs with timestamps [22]. Qiu et al [23] used convolutional neural network joint training by transferring learning across primary cancer sites to achieve great performance in lung cancer and breast cancer classification tasks. However, these complex and efficient models tend to be black boxes and lack interpretability [24] and transparency, which makes doctors reluctant to accept them. Fortunately, in the papers we reviewed, there have been several attempts to solve this problem, such as the application of attention mechanism [25] and Gradient Class Activation Maps algorithm, decision-making process visualization [26]. In addition, some of the papers in this review have developed novel EHR mining algorithms that perform better than baseline algorithms, such as the “semi-supervised set covering machine” [27] and an unsupervised framework of “subgraph augmented non-negative tensor factorization” [28].

#### AI Performance Metrics

In our review, 124 papers used one or more of the precision, sensitivity (recall), specificity, F-score, accuracy, and AUC to measure the performance of AI model. The AUC was generally high, that is, 0.7 and above. Accuracy ranged from 0.613 to 1. The precision ranged from 0.353 to 0.999, except for the 4 prediction models for CRC reported by Kop et al [29], Hoogendoorn et al [30], Hong et al [31], and Birks et al [9], wherein their models had precision less than 0.1. Kop et al [29] and Hoogendoorn et al [30] also reported the lowest F-score of 0.058 and 0.074 in this survey, while Ping et al [32] reported the highest F-score of 0.996. Of the papers reporting sensitivity or specificity, 87% had a sensitivity or specificity greater than 0.7 and more than 50% had a sensitivity or specificity greater than 0.9. 

### Application in the Field of Cancer Care

**Figure 4:**
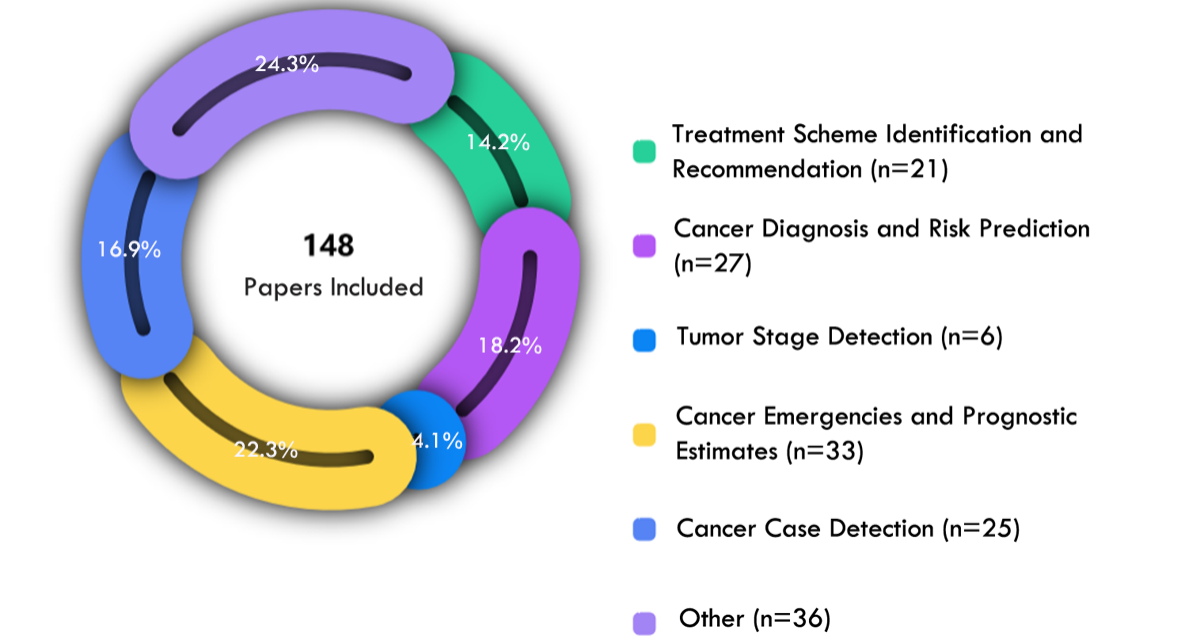
Papers related to artificial intelligence application in cancer care.

AI based on EHRs has permeated the whole cycle of cancer care. The significance of the included papers in the journey of cancer medical care can be broadly divided into several applications. The proportion and number of papers showing the application of AI in cancer care are shown in Figure 4. In this section, we summarize the representative papers.

#### Cancer Diagnosis and Risk Prediction

Of the 148 studies, 27 (18.2%) explored the risk factors for cancer, developmental risk prediction models, and differential diagnosis of cancer, maintaining an AUC of 0.7 and above. In the data of 25,430 patients in the United Kingdom, full blood count indicators were added on the basis of age and sex to predict risk of CRC, and it was found that the AUC of the prediction model (based on logistic regression algorithm) at 18-24 months before diagnosis could reach 0.776 [9]. The prostate-specific antigen density, transversal diameter of the prostate, and other variables were used to establish the decision tree model (the variable with maximum gain was selected as the split variable; other hyperparameters used the default settings) to differentiate prostate cancer from benign prostatic hyperplasia [33], achieving a precision of 0.86.

#### Tumor Stage Detection

Of the 148 studies, 6 (4.1%) used AI to identify explicit and implicit stage information from unstructured EHRs. The performance metrics values of the reported AI models were greater than 0.66. It took less than 1 hour to extract cancer summary stage information from more than 750,000 documents that required a human reader months to years to digest [34]. Two papers explored the staging of lung and prostate cancer with reference to the American Joint Committee on Cancer staging system. Three studies on liver cancer staging used American Joint Committee on Cancer, Barcelona Clinic Liver Cancer, and Cancer of Liver Italian Program staging system.

#### Treatment Scheme Identification and Recommendation

Of the 148 studies, 21 (14.2%) used AI to adapt doses in antidrug regimen [35], assess effect and combination of dose, evaluate cancer therapeutic procedures, and recommend treatment schemes based on EHRs. The precision, recall, specificity, F-score, accuracy, and AUC were above 0.67, except in a model for drug repurposing reported by Wu et al [36]. Savova et al [37] tried to mine endocrine breast cancer drug treatment patterns by combining information extracted from clinical free text through NLP with structured data, and they obtained high specificity above 0.96 for all categories. Goldbraich et al [38] applied NLP techniques to characterize deviations from clinical practice guidelines in adult soft tissue sarcoma across thousands of patient records, identified that approximately half of all treatment programs deviated from the clinical practice guidelines, and analyzed reasons that may reflect the physicians’ rationale in deviation cases. The Oncology Expert Advisor [39] was designed to recommend treatment options by developing a learning model to predict appropriate therapy options for lung cancer with a recall of 0.999, precision of 0.88, and ability to accommodate addition or changes to the approved therapies list.

#### Cancer Case Detection

Of the 148 studies, 25 (16.9%) proposed AI methods to identify patients with specific cancers such as prostate cancer and breast cancer. The AUC was high above 0.9. Features were extracted from progress notes and pathology reports by NLP, which were used to train the SVM model to identify the group of patients with contralateral breast cancer, obtaining an AUC score of 0.93 (hyperparameters were tuned by 5-fold cross-validation) [40]. The accumulation of EHRs and the development of AI have made it possible to have a large cohort study for different clinical problems. Data-driven intelligent approaches, rather than manual chart review, were important for capturing special cases of cancer among a large cohort efficiently.

#### Cancer Emergencies and Prognostic Estimates

Of the 148 studies, 33 (22.3%) focused on extracting tumor prognostic factors, predicting outcomes in individual patients with cancer and developing emergency prediction models for emergency visits and hospital admissions and so on. All reported AUCs were greater than 0.72. Gradient tree boosting model [41] was developed to predict emergency visits and hospital admissions during radiation and chemoradiation based on synthesizing and processing EHRs (demographics, drug therapy, etc) with an AUC of 0.798 (hyperparameters were tuned by 5-fold cross-validation). Regarding the prediction of cancer relapse, patients [42] with childhood acute lymphoblastic leukemia were classified into different relapse risk-level groups by random forest algorithms based on EHRs (white blood cell count, hemoglobin, etc) with an AUC of more than 0.9. For the prediction of cancer survival, breast cancer–related variables, tumor characteristics, and patient demographics were used to developed SVM models (the soft margin parameter C of SVM was selected through cross-validation) to estimate the patient’s survival status of the 3 time periods. AI models were slightly better than the performance of the clinician panel [43]. Compared with traditional methods for survival analysis, AI methods focused on the prediction of event occurrence, applied to high-dimensional problems usually, and showed improvements in predictive performance [44].

### Data and Data Sets

Most papers described experiments conducted on non–publicly available data sets, and more than half of the papers were based on data from a single health care institution, as detailed in Multimedia Appendix 1. Less than 10% of the included papers (n=12) made use of publicly available data sets, that is, SEER, Informatics for Integrating Biology and the Bedside, and Medical Information Mart for Intensive Care data set. A few studies combined clinical practice guidelines, a literature corpus, administrative data, and other types of data on the basis of using EHRs. Focusing on the patient sample size used in the actual study and eliminating the remaining 35 papers that were not specified, 42 had fewer than 500 samples, 17 had between 500 and 1000 samples, and only 18 had over 10,000 samples. Regarding the language used in EHRs, 100 papers exploiting EHRs in English topped the list, followed by papers with EHRs in Chinese (n=18). Algorithms for English report processing have been relatively effective and can be scaled to other languages. For example, an NLP algorithm automatically extracting carcinoma and atypia entities from English pathology reports achieved an accuracy of 0.9 [45]. It was later applied to Chinese breast pathology reports. In comparison with using English reports, this paper [46] discussed the performance of the model and demonstrated that it worked just as well for Chinese processing. Regarding the nature and challenges of EHRs used in the experiment, nearly half of the studies explicitly used only unstructured data such as pathology reports, progress notes, discharge notes, and radiology reports.

## Discussion

### Principal Findings

Of 1034 studies, 148 were selected for the systematic review. Our systematic review has shown that the use of AI to process EHRs has broad applications in providing insights into cancer care, particularly for cancers of female organs, digestive organs, respiratory organs, and intrathoracic organs. ML was the common implementation of AI based on the EHRs of patients with cancer. SVM and logistic regression were the most used ML classifiers. Traditional ML algorithms moved from stand-alone predictions to benchmarks for new approaches. Ensemble methods and DL are on the rise and improving performance. However, the interpretability of complex algorithms is a key issue, and more research is needed on this issue. The results show that most AI models can usually achieve a performance metric value of 0.7. It is worth noting that the CRC prediction models reported in 4 papers had significantly lower precision and 2 of them had lower F-scores. Further investigation revealed that in the design of the experiment, the researchers consciously traded higher false-positive rates for fewer patients that were missed because they believed that the cost of a normal person being wrongly predicted was lower than the cost of missing a patient depending on the characteristics of CRC. However, high false-positive rates would also make medical procedures too costly or invasive and should be analyzed according to the disease investigated. Cancer care could benefit from AI based on EHRs through cancer emergencies and prognostic estimates, cancer diagnosis and prediction, tumor stage detection, cancer case detection, and treatment pattern recognition. The topic of emergency and prognostic estimation had the most research. Finally, we discussed EHRs and databases. Our review found that the vast majority of studies in this area were based on private databases within the institution, resulting in poor portability of the proposed methodology process. Public databases were underused, and few patient records were included in the actual studies. In another way, it also reflects the fact that public databases are still scarce. English EHRs are mainly used, and the exploration of EHRs in other languages is limited. Of course, this may be a bias caused by our selection of English papers only. Fortunately, the existing literature also showed that the processing methods of EHRs in English are relatively mature, and these methods may be transplanted to data in other languages. Much cancer information are stored in unstructured formats of EHRs and are difficult to mine, thus requiring better algorithms and more efforts. Furthermore, EHRs can be combined with other data sources to support AI for cancer care.

### Comparison With Prior Work

Recently, several systematic reviews related to EHRs have been published, with particular attention given to the implementation of EHR systems [47,48]. Several studies have discussed different applications of technology to EHRs, such as blockchain [49]; yet, few have focused on the specific secondary use of EHRs, such as the role in reducing unwarranted clinical variation [6] and patient identification and clinical support in palliative care [50], with even fewer focusing on specific disease areas such as diabetes [51]. There is existing work elucidating the state of AI research in cancers [52,53]. However, to our knowledge, none have focused specifically on the combination of EHRs and AI in cancer, which makes it difficult to have a specific understanding of the current implementation and challenges of this field.

### Limitations

This review examined nearly 12 years of literature and may have the following limitations. First, despite efforts to develop a systematic and careful search strategy, there is no guarantee that all relevant literature will be included. Our search was limited to published literature in English, but searches in other languages or gray literature may provide additional findings. Second, the popularity of EHRs and the degree of data development vary in different countries and environments, which may lead to inconsistency in the quality of the included literature research, and the algorithms and effect evaluation analysis may have an impact. Third, we only considered the literature and did not investigate the AI products in the market. This may need to be further supplemented.

### Conclusions

Our review shows that AI based on EHRs performed well and can be useful for cancer care in 4 areas: categorization of neoplasms, methods and algorithms, application in the field of cancer care, and data and data sets. Based on our review, we propose the following recommendations for future research:

1. The development of new AI methods: The use of hybrid approaches could improve the performance of AI models. DL and ensemble methods have great potential in cancer care. The interpretability of methods must be given more attention. In addition, the models need to adjust the evaluation of performance appropriately according to the disease under study so that it can achieve better practical results.
2. EHR sharing and fusion: There are too few open data sets available for researchers, and the lack of a large annotated gold standard library has become a major bottleneck for research in this field. In the case of complying with data ethics, the sharing of EHRs and multiagency participation in EHR databases is urgently needed. Guidelines, literature data, and corpora in other fields can play an important role in addressing this problem. At the same time, EHRs could be complemented by guides, literature, and corpora in other fields to enhance the benefits of AI.
3. Passion and support from cancer specialists: Recognition and acceptance by practitioners in the fields of cancer care is necessary for the research results to be translated to practice. This requires more human experts in this field to overcome the natural resistance of traditional views, participate in the formulation of a gold standard, reasonably adopt research conclusions, and take responsibility for the actual medical outcomes.

## Appendix

Multimedia Appendix 1 Complete list of the reviewed papers.

## Abbreviations

AI: artificial intelligence
AUC: area under the curve
CRC: colorectal cancer
DL: deep learning
EHR: electronic health record
ML: machine learning
NLP: natural language processing
PRISMA: Preferred Reporting Items for Systematic reviews and Meta-Analyses
SVM: support vector machine
